# Abstracts of papers presented at Flow Analysis VI: Toledo, Spain (8-11 June 1994)

**DOI:** 10.1155/S1463924695000058

**Published:** 1995


Journal of Automatic Chemistry, Vol. 17, No. 1 (January–February 1995), pp. 31–40

### Abstracts of papers presented at Flow Analysis VI: Toledo, Spain (8–11 June 1994)

The VI Flow Analysis meeting was recently held in Toledo, Spain. The organizers of this conference, Professors Valcarcel and Luque de Castro, have kindly allowed the *Journal of Automatic Chemistry* to publish the abstracts of the papers presented. As can be seen from these abstracts, the meeting covered a range of disciplines and applications and was the forum for some interesting discussions and debate. The opening lecture, presented by one of the original pioneers of the flow analysis technique, Professor Ruzicka, was particularly stimulating and set the standard for the meeting.

The ambience of Toledo was ideal for the scientific proceedings and the organizers are to be congratulated on bringing together the world's experts in this area.

**Peter B. Stockwell**
*Editor*

### Flow injection bioanalysis—from sample to a living cell

*Jaromir Ruzicka, Department of Chemistry, BG-10 University of Washington, Seattle, WA 98115, USA*

While in the past the development of flow injection was focused mainly on automation of traditional reagent chemistries and solution handling tasks, the next decade should bring novel techniques and should lead to the discovery of further, unique areas of application. In this way analytical science will provide novel tools to its own community, and to biology, biotechnology, chemical processing and environmental studies. The author supported this viewpoint by discussing two emerging techniques: flow injection cytoanalysis (FIC) and flow injection on renewable surfaces (FIRS). He demonstrated how the second of these two methods became possible through the advances of the first one, and he speculated on the impact FIRS might have on such different areas as biotechnology, immunoassays, sensor technology and chromatography.

### New developments in flow injection immunoassays

*G. S. Wilson, P. C. Gunaratna and T. L. Hendrickson, Departments of Chemistry and Pharmaceutical Chemistry, University of Kansas, Lawrence, KS 66045, USA*

The majority of immunoassays are carried out in microtitre plates or tubes, configurations which are far from optimal. Such assays depend upon the chemisorption of an antibody or antigen on the wall of the container. The surface area is not very large and can accommodate only about $10^{-12}$ mol/cm$^2$ of adsorbed antibody. (In a typical microtitre plate this corresponds to about $10^{-12}$ mol.) Moreover, the extent of adsorption from well to well can vary by more than 100% and under assay conditions antibody can desorb. These properties can lead to both low dose and high dose 'hook effects'. In addition, the format is not conducive to automation nor to careful control of reaction conditions. By contrast, flow injection immunoassays can be carried out using the same instrumentation used for HPLC, though the high pressure capability is not needed.

It has been widely assumed that immunochemical reactions must run to completion, leading to incubation times of hours to days. However, reaction between an immobilized antibody and a soluble antigen in a flowing stream can proceed to completion at nearly diffusion-controlled rates. If the reaction conditions are carefully controlled, then it is not necessary to have complete reaction.

Two recent examples were discussed. The first involves the development of an immunoassay for α-difluoromethyl ornithine, an anticancer agent that irreversibly inhibits the enzyme ornithine decarboxylase. The analyte is essentially a fluorinated amino acid and would normally be analysed by a competitive binding assay. The authors have developed a non-competitive method which has virtually all of the advantages of the sandwich assay: extended dynamic range and improved sensitivity. Basically a 'back titration' method, it takes advantage of non-equilibrium measurements in a flowing stream. The second example involves the analysis of enkephalins, small neuroactive peptides. Endogenous enzymes can degrade the sample thus complicating the analysis. Thus flow injection not only provides an analysis method, it also provides rapid on-line sample preparation. These assays also depend on the preparation of high quality antibodies and this important subject was also discussed.

### Electroosmosis as a pumping means in miniaturized flow analysis: FIA, SIA and beyond

*Purnendu K. Dasgupta, Department of Chemistry, Texas Tech University, Lubbock, TX 79409-1061, USA*

The fluid delivery system remains the most difficult component to miniaturize in implementing flow analysis schemes in a micro scale. It is possible to use electroosmosis in such applications as an inexpensive, highly reliable pumping mechanism with high reproducibility and no moving parts. By hydraulic coupling, it is possible to pump fluids that cannot be directly exposed to a high electric field. Applications were described that encompass single or multichannel flow injection analysis, sequential injection analysis using a multiport valve, on-line preconcentration etc.

### Process flow injection analysis of liquid and gas streams in the chemical and petrochemical industries

*Don C. Olson, FIA Solutions, P.O. Box 670786, Houston, TX 77267-0786, USA*

Process flow injection analysis began to emerge only a few years after the first publication on FIA in 1975 by J. Ruzicka and E. H. Hansen. The pioneers of process FIA came from the chemical and petrochemical industries in the USA, but the technology has since spread worldwide to the biotechnology, mining, paper and pulp, brewing, dye, refining, and nuclear industries. Its use covers a broad range of on-line, at-line, and off-line applications.

A survey of the use of process FIA in the chemical and petrochemical industries was presented. The techniques and technology important to process FIA were covered, as well as their strengths, benefits, and pitfalls. A new generation of the technology, process sequential injection analysis, was also described.

### Mathematical modelling of flow injection systems

*Spas D. Kolev, Faculty of Chemistry, University of Sofia, 1 James Bourchier Avenue, BG-1126 Sofia, Bulgaria*

Although the principles on which flow injection (FI) analysis is based are well understood and extensively used in the analytical practice, the development of its theoretical foundations is far from completed. The main reasons for this are the complexity of the phenomena (for example diffusion, convection, chemical kinetics) taking place in FI systems and the variety of FI configurations (for example single and multi-line systems with one or several confluence points, mixing chambers, dialysis and gas-diffusion modules, open–closed systems) and modes of operation (stopped, reversal, and sinusoidal flow). Regarding the interconnections between their flow-through elements, the FI manifolds can be considered as composed of two basic flow configurations, i.e. the single-line and the conjugated two-line system. Numerous mathematical models employing ideas from different scientific areas (for example statistics, chemical engineering, artificial intelligence, chromatography) have been developed. A classification incorporating all these models and based on the main principles on which they are built is required for allowing an impartial comparison between them. It will also help a researcher in choosing an appropriate model with respect to the concrete FI system to be modelled and the aims of the modelling. According to the way the existing models view a FI system, they fall into two main groups: 'black box' and 'analytical-experimental' models. The former models assume that an FI system can be described only on the basis of its input–output relationships. These models can be subdivided further on with respect to the specific approach (for example regression analysis, artificial neural networks, statistical moments, impulse-response functions, some special functions) that they utilize. The analytical–experimental models consider the real processes taking place in FI manifolds and could be deterministic or stochastic (for example random walk simulation) in nature. The former models include lumped (for example cell models) and distributed (for example dispersion models) parameter models. The applicability of the mathematical models mentioned above for the description of single-line and conjugated two-line system has been examined. The axially-dispersed plug flow model offers an acceptable compromise between the contrary requirements for mathematical simplicity and precision.

### Electrogenerated chemiluminescence detection in flowing streams

*T. A. Nieman, Department of Chemistry, University of Illinois, 600 S. Mathews Avenue, Urbana, IL 61801, USA*

Chemiluminescence (CL) reactions are commonly used in combination with flow streams. Flow injection analysis (FIA) is used to characterize CL systems and CL is used for detection in assays based on FIA, HPLC, and capillary electrophoresis. In most analytical applications of CL, the reaction is initiated by addition of a solution of a key reagent. However, with electrogenerated chemiluminescence (ECL) the CL reaction is initiated at an electrode either by direct reaction of the CL reagent or by electrogeneration of some necessary reactant. In addition to reducing the number of reagent solutions that must be pumped and mixed, ECL provides spatial and temporal control via the electrode potential's ability to turn the reaction 'on' and 'off'. Modulation of the ECL emission is possible for fundamental characterization and practical application.

Characteristics and applications of electrogeneration of CL with several reactions were presented: luminol (for determination of hydrogen peroxide and determination of species labelled with a luminol moiety), acridinium esters (used as immunoassay labels), aryl oxalates (peroxyoxalate CL for determination of fluorophores), and tris(2,2′-bipyridyl)ruthenium(II), or $Ru(bpy)_3^{2+}$ (for determination of oxalate, amino acids, NADH, and aliphatic amines).

The $Ru(bpy)_3^{2+}$ system is especially interesting because the original CL reagent is regenerated during the reaction. Thus, there is considerable utility in immobilizing the $Ru(bpy)_3^{2+}$ on the electrode surface. Several different methods of immobilizing $Ru(bpy)_3^{2+}$ have been investigated and the characteristics of these forms for CL detection in FIA and HPLC applications were presented. Use of solution and immobilized $Ru(bpy)_3^{2+}$ was compared, and sensing systems combining immobilized dehydrogenase enzymes with immobilized $Ru(bpy)_3^{2+}$ were described.

### Flow injection and electrochemical analysis—

*Wolfgang Frenzel, Institut für Technischen Umweltschutz, Fachgebiet Luftreinhaltung Technische Universität Berlin, D 10623 Berlin, Germany*

The application of electrochemical detection principles in flow injection analysis (ED-FIA) enjoys continued popularity. This presentation described a survey of important contributions and emphasized the advantages and limitations of employing ED-FIA. Instrumental developments with respect to flow-cell design and applications in various fields were critically reviewed.

Particular attention was focused on 'unique potentialities' offered to both, the *Electroanalysist* and the *FIAist* applying flow injection methodology and electrochemical detectors, respectively. It was shown that on the one hand improved performance of electrochemical sensors is generally achieved under FI-conditions, and, on the other hand, electrochemical detectors give access to otherwise difficult analysis problems.

### Development of an FIA system with on-line microwave enhanced reactions for inorganic and organic analyte preparation

*Stephen J. Haswell, School of Chemistry, University of Hull, Hull HU6 7RX, UK*

The use of microwave energy as a controlled heating source for chemical reactions has in recent years found important applications in analytical science. The majority of such applications have focused exclusively on the use of microwave ovens or tuneable cavities for thermally assisted acid decomposition of inorganic and organic matrices. To a lesser extent, chemical derivatization of analytes, synthesis, catalysis and extraction reactions have also been reported in the literature. Almost without exception, all of the current methodology using microwave energy has been based upon batch or discrete reaction configurations; this paper, however, described the emergence and early use of on-line microwave enhanced reactions in which a flow injection methodology has been adopted. The paper briefly reviewed the current status of the technique and then described specific examples of systems developed in the author's laboratory. These systems included on-line matrix decomposition, hydrolysis, saponification and trans esterification reactions coupled to a range of detector types for a selection of different analytes. The paper considered the many problems associated with interfacing sample preparative procedures with measurement systems, with particular reference to matrix interferences and calibration. The paper also considered the mechanisms occurring under a FIA regime which offer a more efficient and selective reaction system compared to the open or closed vessel type. The paper concluded with a discussion of possible future applications for highly automated on-line microwave enhanced chemical reaction systems.

### Discontinuous flow analysis: generation of fluid flows by differential pumping

*G. R. Scollary, School of Chemistry, The University of Melbourne, Parkville, 3052, Australia*

The flow technique of Discontinuous Flow Analysis (DFA) was designed and developed by the Australian instrument company, Ionode Pty Ltd. DFA was first described in the scientific literature in 1989, although its conception and patent applications date back to 1985.

This paper described the design of the DFA instrumentation, identifying the significant differences between DFA and other flow based analytical techniques. The use of differential flow as a means of sample introduction and the requirement for near-perfect radial mixing was emphasized. Means for the production of a quantitative relationship between detector response and the sequence of flow rate ratios of sample to reagent were outlined. The ease with which various flow profiles can be established by DFA was stressed by describing cam and piston designs for titration, incremental and linear gradient profiles.

Applications of DFA were reviewed. The feasibility of DFA being used as a means of sample presentation to other analytical methods, such as flame AAS and ICP, was outlined and the significant advantage of achieving both calibration and sample analysis in the one cam cycle was highlighted. Future developments of DFA which will address the three major issues in modern analytical chemistry of absolute analysis, process monitoring and real time quality assurance of the analytical measurement were discussed.

### The development of flow injection atomic spectrometry as a product line

*B. Welz, Department of Applied Research, Bodenseewerk Perkin-Elmer GmbH, D 88647 Uberlingen, Germany*

The author's research group stumbled over FI during a search for a way to automate the hydride generation (HG) technique for atomic absorption spectrometry (AAS). It not only worked well but performed much better than expected. The same detection limits from 500 μl of sample were obtained as with 50 ml which was previously required, an improvement by two orders of magnitude in absolute terms. Similarly reagent consumption was dramatically decreased and the sample throughput increased. Research efforts were then turned to flame (F) AAS applications, such as microlitre sample volumes, samples with high dissolved solids content, automatic addition of buffers and automatic dilution. It was discovered that all these features could be included in the new system without any hardware changes, so the new accessory for HG and FAAS could be introduced at the same time. The next step was on-line preconcentration on packed microcolumns; this started with FAAS and the experience was transferred essentially without modification to electrothermal (ET) AAS. For that technique FI opened a new world of applications, decreasing detection limits to the low ng/l-range without increasing contamination problems. This on-line preconcentration made differential determination of individual oxidation states of an element possible. On-line microwave digestion procedures were also investigated, and an algorithm for interference recognition and correction was developed, expanding the use of FI from sample pretreatment to data handling. That brought the author's group close to realization of the dream of a fully automated analyser that takes an untreated sample and delivers the validated result.

### Dual valve flow injection system with FTIR detection for the determination of hydrolytic enzyme substrates

*R. Kellner, B. Lendl and F. Rosenberg, Institute of Analytical Chemistry, Vienna University of Technology, Getreidemarkt 9/151, A 1060 Vienna, Austria*

The versality of flow analysis with different kinds of detectors has been demonstrated over the past two decades of use. Despite its potential for the direct qualitative and quantitative multi-component analysis of organic substances due to the specificity of the IR absorption bands and the possibility of simultaneously acquiring information over the whole spectral range, the Fourier transform infrared (FTIR) spectrometer has found only very limited use as detector in flow injection analysis.

The combination of an FIA–FTIR system incorporating enzymatic reactions to determine glucose and urea simultaneously and in complex matrices has previously been described by the authors. Although the compensation of background absorption was possible, a double injection was necessary in order to acquire a background spectrum (before reaction) and a sample spectrum (after reaction). This inconvenience is eliminated by applying a dual injection valve manifold. This double-line manifold consists of an injection valve, in the loop of which a second injection valve is integrated. By switching this second injection valve, the injected sample can either be passed through the enzymatic reactor which is located in the loop of the second valve, or it is bypassed. As a result, the sample plug consists of a leading zone of the unreacted sample (which has not been passed through the reactor) and a tailing zone where conversion has taken place in the enzymatic reactor. Thus, a spectrum can be obtained from both the reacted sample and the background using a single injection. Data acquisition can be carried out both in the stopped flow and the continuous flow mode. Stopping the flow during data acquisition improves the signal-to-noise ratio by increasing the number of scans.

Using this double valve manifold, the reactions of hydrolytic enzymes such as the urease, invertase or oligo- and polysaccharide hydrolyases have been investigated. Quantitative analysis of urea, oligo- and polysaccharides in the mmol per litre range is possible and examples were presented for the application of this system to process control in the food industry.

### Biosensor development for environmental analysis of phenols and related compounds

*E. Dominguez, F. Ortega, Department of Analytical Chemistry, Faculty of Pharmacy, University of Alcalá de Henares, E 28871 Alcalá de Henares (Madrid), Spain; L. Burestedt, J. Emneus, L. Gorton, G. Marko-Varga, Department of Analytical Chemistry, Lund University, PO Box 124, S-22100 Lund, Sweden; M. Lutz, H. Irth, Division of Analytical Chemistry, Leyden University, Gorlaeus Laboratories, PO Box 9502, 2300 RA Leyden, The Netherlands; D. Puig and D. Barcelo, Department of Environmental Chemistry, CSIC, Jordi Girona 18-26, E 08034, Barcelona, Spain*

Natural organic compounds including phenols and related compounds are widely distributed throughout the environment. Phenols and substituted phenola are products of many industrial processes, for example the manufacture of plastics, dyes, antioxidants and waste waters from the pulp industry. The increasing demand for the characterization and quantification of phenolic compounds present in environmental water samples and screening of a large number of samples with complex matrix composition has stimulated progress with new detection principles in this area.

This presentation concerned the strategies which are involved in a project approved by the Commission of the European Communities for the selective determination of phenols and related compounds in environmental water samples. The aim is to develop an on-line system including sample pretreatment and detection. This is achieved by coupling an amperometric biosensor to an automatic liquid chromatographic system for sample clean-up and separation. The challenge in the development of this integrated system is the construction of an interface between sample handling and biosensor technology which guarantees the optimal performance of both processes. Results on the on-line coupling of various types of tyrosinase based biosensors to the sample pretreatment unit were presented. These results were compared with standard LC-MS methods for validation of the catalytic detection system.

### Dynamic analysis of binding process of protein on glutaraldehyde-activated support

*H. Ukeda, T. Ishii, M. Sawamura and H. Kusunose, Department of Bioresources Science, Faculty of Agriculture, Kochi University, Monobe B-200, Nankoku 783, Japan*

Glutaraldehyde (GA) has been extensively used for the immobilization of enzymes onto an amino-terminal support. In this study, based on the FIA method, a dynamic analysis of the binding process of protein onto the support activated with GA was performed in order to clarify the chemistry of the immobilization reaction. A solution of bovine serum albumin (BSA) was repeatedly injected into a column packed with aminopropyl-controlled pore glass activated with GA (GA-CPG) at appropriate time intervals. The experimental elution profile of BSA from the column was monitored with a UV detector and analysed by a model based on the assumption that two modes are involved in the binding of BSA onto GA-CPG. Binding process parameters, such as bound amount and binding rate constant, were determined by the curve-fitting method. The increase of ionic strength of the carrier solution reduced the total bound amount of BSA, suggesting an involvement of ionic interaction in the binding. The maximum bound amount was recognized at pH 6 with a carrier of low ionic strength, and at pH 7 for a higher ionic strength carrier. While reduction of GA-CPG with sodium borohydride remarkably decreased the bound amount, blocking treatment with amine did not lower the amount. This may mean that a functional group on the support, other than an aldehyde, is responsible for the binding of BSA. The bound amount of BSA at the restricted diffusion sites was noted to increase with rise in pH during the activation step with GA prior to the binding process, whereas there was no significant difference in the total bound amount. This implies that aldol condensation of GA in alkaline pH could result in changes of the internal pore structure. These results show that FIA can make a significant contribution to designing a bioreactor.

### Crystal seeding in flow injection turbidimetry: determination of total sulphur in plants

*Sandra M. B. Brienza, José A. Gomes Neto, Instituto de Física e Química de S. Carlos, Universidade de São Paulo; Raquel P. Sartini and Elias A. G. Zagatto, Centro de Energia Nuclear na Agricultura, Universidade de São Paulo, 13416-000 Piracicaba SP, Brazil*

Addition of a reproducible suspension in order to increase supersaturation conditions is a powerful approach to flow turbidimetry. This crystal seeding permits a simplification in system design, and an improvement in sampling rate and/or sensitivity in flow based methodologies which are usually limited by rate of turbidity increase.

The feasibility of the approach was demonstrated in this presentation—a turbidimetric flow-injection procedure was developed for determination of total sulphur in plant materials based on lead sulphate formation after adding a confluent stream consisting of a lead phosphate suspension. Effects of ethanol content, reagent concentrations, acidity and timing were investigated. Up to 600 samples can be run per hour, and no special procedures for system washing are needed. Surfactant is not required and no baseline drift is noted after 8 h operation periods. Results are precise (r.s.d. $< 0.005$) and in agreement with those obtained by the usual flow-injection procedure based on barium sulphate.

### Combined separation and sample introduction in flow analysis

*Wolfgang Künnecke, Jochen Mohns and Ursula Bilitewski, Gesellschaft für Biotechnologische Forschung (GBF) mbH, Project Group Biosensors, Mascheroder Weg 1, 38124 Braunschweig, Germany*

Biochemistry-based analytical systems, and especially biosensors, suffer from a relatively low operational stability which is a limiting factor for routine use. The incorporation into flow systems enables a high degree of automation and simplifies data evaluation. Consequently, fast recalibration becomes possible and these combined systems are attractive to analysts.

However, there is still a serious problem of sample separation. Well known examples are fermentation and *in vivo* monitoring. Both applications have similar requirements: sterile or aseptic separation of the analyte being the most important of them. Another important factor is sample volume. Sterile separation is often performed using dialysis. Examples are the tubing method in fermentation technology and microdialysis for in-vivo measurement.

There are three chief advantages to integrating separation and sampling into one device: first, the system becomes very simple, i.e. an injection valve is dispensable. Second, almost no volume is extracted from the medium. Third, time based sampling is more flexible compared to volumetric sampling using injection valves. In the first case, different analyte concentrations can be accumulated due to variations in sampling time, whereas the latter method requires a manual exchange of the sample loop of an injection valve. Examples for fermentation monitoring of glucose and microdialysis sampling for future *in vivo* studies were presented to demonstrate the flexibility of this new flow-technique. Flow-Diffusion Analysis (FDA) is the term chosen for this technique.

### Tandem (hyphenated or coupled) flow injection systems—a new dimension in flow injection analysis

*Jacobus F. van Staden, Department of Chemistry, University of Pretoria, Pretoria 0002, South Africa*

The first applications of flow injection analysis as analytical technique were relatively simple, but very successful systems. This was a valuable and timely concept that filled a gap in chemical analysis in the mid 1970s. The main purpose was to supply a simple and cheap, but efficient robust analyser for faster analysis. At that stage the principle components of the first systems were mainly sample preparation with sample injection and processing in a manifold and detection of the processed sample zone. Gradually the systems become more complex and were extended to include other analytical techniques and concepts. The integration of flow injection analysis with multifunctional analytical techniques is a reality of the future which eventually will culminate into useful process analysers. There are already a number of tandem, hyphenated or coupled flow injection systems that became available to analytical laboratories. These concepts bring a new dimension into flow injection analysis. The various configurations were discussed and some systems highlighted.

### *In situ* monitoring of nutrients (nitrogen and phosphorus) in marine, freshwater and terrestrial environments

*Paul J. Worsfold, Trevor McCormack, Anthony R. J. David and Nick J. Blundell, Department of Environmental Sciences, University of Plymouth, Plymouth, UK*

FI techniques are well established for laboratory applications and are being increasingly used for process analysis. Their potential for remote environmental monitoring, however, has yet to be realized. This presentation discussed the design, construction, deployment and validation of FI based monitors (incorporating solid state spectrophotometric detection) for the determination of nutrients, particularly nitrate, in a range of environmental situations. Particular attention was paid to the interface between the environmental compartment (for example, estuarine water, soil pore water) and the monitor, the long term reliability of the individual monitor components (including reagents) and the environmental relevance of the data obtained.

The performance requirements, for example, length of deployment, frequency of analysis, limit of detection, depend on the specific application. One of the aims of the present work is to deploy intelligent submersible instrumentation that can routinely relay analytical and diagnostic information back to a central facility. For example, a number of low cost systems could be deployed along an estuary to monitor event triggered (for example, storms) and seasonal variations in nitrate fluxes. This paper reported on the progress made to date towards this objective.

### On-line flow system for automatic monitoring of fermentations based on microdialysis sampling, flow injection/column liquid chromatography and amperometric biosensing

*L. Gorton, Department of Analytical Chemistry, University of Lund, PO Box 124, S-221 00, Lund, Sweden*

In fermentation technology there is a need for rapid and automated monitoring of the content and changes in composition of the fermentation broth to increase the knowledge of the process and for feedback control. Microdialysis is a well-established technique in medicine for *in vivo* monitoring but has only recently been studied as a means to sample fermentation processes in an aseptic and representative way. Sampling is based on self-diffusion of analytes and will not affect the volume of the fermentation process, thereby making microdialysis suitable for long-term studies of laboratory-scale fermentations, in contrast to established sampling techniques based on continuous removal of sample volume from the fermentor. The recovery of analytes from the broth will be affected by the choice of membrane, size and design of the probe, and the flow rate of the perfusion medium. Various probes with different membranes, designs, and sized were recently investigated in model systems and in real fermentation media.

After sampling, the perfusion medium is transported to fill an injecton loop connected either to a flow injection system or a column liquid chromatographic system using a flow through electrochemical detector cell. The sensing electrode, operating at −50 mV vs. Ag/AgCl, is based on a carbon paste electrode chemically modified with a hydrogen peroxide producing oxidase co-immobilized with peroxidase. Improved sensor characteristics (long-term stability and increase of response) are obtained through addition of activators/stabilizers to the carbon paste. The electrochemical cell can be constructed to incorporate several sensing electrodes with different enzymes to produce an array type detection unit enabling simultaneous, selective, and sensitive monitoring of substrate depletion and product formation.

### The role of atomic fluorescence detectors for speciation studies on mercury, arsenic and selenium

*Peter B. Stockwell and W. T. Corns, P S Analytical Ltd, Arthur House, B4 Chaucer Business Park, Watery Lane, Kemsing, Sevenoaks, Kent TN15 6QY, UK*

While current legislative requirements are to measure the total elemental content for mercury, arsenic and selenium, the differences in the toxicity of the organo-metallic species make it important to measure these reliably. Since the levels of interest for all species are quite low, atomic fluorescence detectors specifically designed for the task have considerable potential. Chromatographic systems designed to couple to these detectors were described, along with a number of analytical applications.

The systems were quantified by the analysis of certified reference materials.

### Multivariate data analysis of flow injection signals

*B. Hitzmann, T. Pekeler and T. Kullick, Institut für Technische Chemie, Universität Hannover Callinstrasse 3, 30167 Hannover, Germany*

Standard evaluation methods of measurement signals from flow injection analysis just use one characteristic feature of the whole signal to calculate the analyte concentration: all the information of the measurement signal is condensed to the signal's height, integral or width. Therefore, most of the information that is hidden in the contour of the whole signal is lost. But this information can be used to increase the quality of the evaluation result.

The authors presented examples of multivariate data analysis of flow injection measurements and demonstrated how these evaluation methods can be used to increase the sensitivity, as well as the selectivity of the results. Principle component analysis and neural networks were applied in the evaluation. The measurement signals were obtained from various flow injection analysis systems (FI-systems) for the monitoring of glucose, urea and penicillin concentrations. Some of the measurements (obtained from samples with a changing buffer capacity using pH-FET detectors) can only be evaluated by the multivariate methods and not by standard methods such as peak height, integral or width.

A comparison of the requirements and the quality of these evaluations methods for flow injection measurements was given. The possibility of applying this method to other FI-systems was discussed.

### Flow-through cell luminescence sensors for clinically important chemical species

*M. E. Diaz-Garcia, F., Alava, M. J. Valencia, R. Pereiro and A. Sanz-Medel, Department of Physical and Analytical Chemistry, Faculty of Chemistry, University of Oviedo, 33006 Oviedo, Spain*

Immobilization of luminescent reagents to solid materials, packed into flow-through cells, has drawn much attention in recent years for the development of luminescence optosensors due to the important advantages accrued from the coupling of solid surface luminescence, particularly room-temperature phosphoresence (RTP), with flow injection techniques. In this presentation the development and application of the concept was illustrated for fluorescence and RTP detection of cations, gases and antibiotics.

A flow-through type and a fibre optic probe-type sensor based on the fluorescence enhancement of a fluorigenic crown-ether due to potassium concentration in aqueous solution have been developed. A detection limit of 0·4 μμM $K^+$ for the optosensor and of 0·8 μM $K^+$ for the optrode were found. Other advantageous analytical features of such a transducer were discussed. The optrode device has been successfully tested for $K^+$ determination in sera, milks and waters.

RTP metal-Ferron chelates (Al, Ga, Zr) immobilized on a strongly basic-anion exchange resin are proposed as suitable indicators for oxygen RTP-optosensing in gas mixtures, aqueous solutions and organic solvents. The analytical performance characteristics and potential advantages of such RTP oxygen sensor in enzyme mediated reactions were dealt with.

A flow-through optosensor for tetracyclines (TC) based on the transient immobilization on a nonionic resin of the TC-$Eu^{3+}$ chelate and RTP energy transfer detection is also proposed. The analytical performance characteristics of such optosensor fulfill the requirements of analysis of very low levels of TCs in real samples (urine and pharmaceuticals). Simultaneous quantification of three TCs was demonstrated by time-resolved RTP multivariate analysis optosensing.

### Membrane-based gas sensors for the determination of $NO_2$

*D. Schepers, G. Schulze and W. Frenzel, Technische Universität Berlin, D 10623 Berlin, Germany*

Diffusion controlled gas sampling using microporous or homogeneous membranes coupled to continuous flow systems has been reported for the determination of gaseous compounds in the atmosphere. In this method the sample gas is continuously sucked through one channel of the membrane unit, diffuses across the membrane and is trapped in a recipient stream where detection takes place. The method offers real-time monitoring capabilities with high sensitivity. In placing the detector within the sampling-unit, considerable improvement in detectability can be achieved due to the absence of dispersion. This paper reported on the development and application of such kinds of flow-through gas sensors based on different detection principles for the determination of $NO_2$.

In the spectrophotometric variant the acceptor channel of the membrane module is delimited by two optical fibres providing transport of light from the halogen lamp and to the photodiode detector. Halting the flow of the recipient solution leads to concentration of the trapped species accompanied by a steady increase of absorbance which is directly monitored in real time. Evaluation of results is done as in kinetic measurements by fixed-time measurements. This allows the preconcentration time to be adjusted by feedback detector control, i.e. out of range-measurements can be prevented. For determination of $NO_2$ the common Saltzmann reaction is employed. By appropriate choice of the acceptor chemistry and the associated detection wavelength, the photometric gas sensor could be applied to other gaseous species.

Liquid-phase chemiluminescence detection utilizing the reaction between gaseous $NO_2$ and luminol in alkaline solution has also been applied. Here, the photomultiplier is directly connected to the membrane-based gas collection device.

The advantages of this approach are simplified equipment and higher sensitivity.

### Spectrophotometric flow injection determination of metals using indicator-immobilized flow cell

*T. Imato, K. Ishii, K. Saitoh, E. Shiozaki, Y. Kawabata and N. Ishibashi, Department of Chemical Science and Technology, Faculty of Engineering, Kyushu University, Hakozaki, Higashiku, 812 Fukuoka, Japan*

The authors have proposed a spectrophotometric flow injection determination method of metals by using a metal buffer, mixture of ligand and metal-ligand complex, containing an appropriate indicator. The method is based on the fact that the metal injected is distributed equally to the ligand and the indicator in the metal buffer, depending on their composition, because the indicator is selected to have almost the same stability constant of metal-indicator complex as that of the metal-ligand complex. If the indicator can be immobilized to a flow cell of a spectrophotometric detector, the system should be simpler, and an application of this method to optical sensing using an optical fibre is possible. In the present paper, the authors presented fabrication of a flow cell immobilized with an indicator and its performance for determination of metals in the flow injection system.

Two immobilization methods were examined for indicators, Calmagite and Arsenazo-III, for determination of alkaline earth and rare earth metals. The indicators were immobilized inside a tubular anion exchange membrane (Toso Co., Japan) by using sulphonic group of their indicators. Another immobilization method is based on ion pair extraction with trioctylmethylammonium. The extracted ion pair was dispersed in a plasticized poly(vinyl chloride) (PVC) membrane. The resulting membranes were set to the flow cell of a conventional spectrophotometer with some modification. A flow system of two-channels, a carrier stream (water) and a ligand solution stream, were used. Nitrilotriacetic acid and ethylenediaminetetraacetic acid were used as the ligand solution for determinations of alkaline earth and rare earth metals, respectively. Both flow cells constructed by the tubular anion exchange membrane and the PVC membrane could detect metals as peak-shaped signals. However, gradual drift of a baseline was observed for the flow cell constructed by the plasticized PVC membrane. This seems to be due to dissolution of the indicator in the PVC membrane to the flowing stream. The performance of the flow cell was demonstrated in this paper.

### The use of chemiluminescence detection for flow analysis

*Neil W. Barnett, School of Biological and Chemical Sciences, Deakin University, Geelong, Victoria 3217, Australia*

This paper discussed three different chemiluminescent (CL) reactions as detection systems for flow analysis:

(1) The determination of morphine in process streams using the CL emission resulting from the reaction of morphine with acidic potassium permanganate in the presence of tetraphosphoric acid. Interferences from structurally similar alkaloids were negligible due either to poor CL response or low concentrations. The nature of the emitting species was also discussed.

(2) The CL reacion of tris(2,2′-bipyridyl)ruthenium(III) with various reducing agents has been known for many years. By employing this chemistry, a very sensitive flow analysis method for the determination of codeine in process streams has been developed. A detailed investigation of the relative responses of related alkaloids at various pH values revealed that good selectivity could be achieved with a judicious choice of acidity. Some discussion on the mechanism of the reaction was presented.

(3) The so-called 'peroxyoxalate' (PO) CL has been utilized successfully for sensitive detection in reversed phase HPLC for some years. The nature of the energy transfer species is somewhat speculative. By monitoring the background emission from a PO-CL system under various chemical conditions, some insight into the CL mechanism has been gained, together with the spectroscopic nature of the excited states present.

### Determination of hydrogen peroxide in seawater by flow injection and chemiluminescence detection

*David Price, Paul J. Worsfold, Department of Environmental Sciences, University of Plymouth, Plymouth, UK; and R. Fauzi C. Mantoura, Plymouth Marine Laboratory, West Hoe, Plymouth, UK*

Hydrogen peroxide is ubiquitous in the hydrosphere and acts as a key component in a variety of biogeochemical pathways in seawater. It is particularly influential at the sea surface where it is present at concentrations of up to 500 nM. At these elevated levels it can control the redox state of the water and may react with a variety of influential marine chemical species. Hydrogen peroxide is a photochemically produced transient and is highly reactive. The analytical challenge has been to design and optimize a FI monitor capable of rapid analysis onboard ship to prevent decomposition of the analyte.

Chemiluminescence (CL) with a low noise photomultiplier tube (PMT) was chosen as the detection system due to its sensitivity and proven track record at sea. The CL reaction used is the $H_2O_2$ induced oxidation of luminol which is most efficient at pH 10·8 and catalysed by cobalt(II). The FI manifold was designed to be as simple as possible to allow rapid sample throughput (120 injections per hour) and automated using an in-house designed 8052 micro-controller. A Simplex procedure was used to optimize the FI manifold and reagent concentrations for $H_2O_2$ standards prepared in both Milli-Q and sampled seawater. The existence of $H_2O_2$ in reagent solutions was found to produce an elevated CL background which masked the smaller CL signals. Hydrogen peroxide-free source water (Milli-Q) was prepared for reagent solutions by passing through a column of $MnO_2$ coated Amberlite XAD-7 polymeric beads. This standard procedure produced a 100-fold decrease in CL background and led to detection limit of 2 nM in Milli-Q and 10 nM in seawater ($s/n = 3$). The linear range of the method was extended using a four setting pre-amplifier housed with the PMT to allow detection over the range of nM to µM.

Successful shipboard trials were carried out in the western Mediterranean onboard RRS *Discovery* in July 1993. Depth profiles from predominantly oligotrophic stations exhibited surface maxima of up to 150 nM with a rapid decrease with depth to below the detection limit at 60–120 metres. Day–night peroxide casts show a surface minimum just before sunrise and steady increase during the day to a maximum in the late evening. The profiles also demonstrated the capabilities of $H_2O_2$ as a surface tracer after storms and increased wind driven mixing processes.

The results from the development and application of the simple FI based monitor were presented along with future trends for marine applications of FI.

### Determination of iron and zinc in adipose tissue by microwave-assisted mineralization and flow injection graphite furnace atomic absorption spectrophotometry

*J. L. Burguera, M. Burguera, M. Gallignani, M. R. Brunetto, P. Carrero and C. Rivas, Department of Chemistry, Faculty of Sciences, University of Los Andes, PO Box 542, Mérida 5101-A, Venezuela*

Flow injection was used to develop an on-line automated focused-microwave-assisted system for the mineralization of adipose tissue and the determination of iron and zinc by graphite furnace atomic absorption spectrophotometry. Dissolution and mineralization of 20–30 mg of samples occur by its alternative exposition to the irradiated zone while a sulphuric and nitric acid mixture flows through the sample tip. Additional flows of Triton X-100 and Pd-Mg matrix modifier were introduced to avoid detrimental accumulation of solids on the wall of the tubing and to minimize matrix interference effects, respectively. Selected aliquots of mineralized samples were introduced with a sampling arm assembly, by means of air displacement, into the graphite furnace. The entire system was controlled by a computer, independent of the spectrometer. The analysis of adipose tissue containing ca. 5.4 and 4.3 $\mu g\ g^{-1}$ of iron and zinc, respectively, with good precision and accuracy was possible. The main advantage of the method is that the determination of iron and zinc in adipose tissue is performed in a totally closed system with a minimum of sample manipulation, exposure to the environment and minimum operator attention.

### On-line digestion and filtration for determination of total phosphorus in anhomogeneous environmental samples

*I. D. McKelvie, R. Sthapit, R. L. Benson and B. T. Hart, Water Studies Centre and Chemistry Department, Monash University, Caulfield East, Australia*

Total phosphorus (TP) concentration is an important parameter for water quality assessment and process control in wastewater treatment. Traditional batch methods for determination of total phosphorus involve extensive digestion with acid and oxidant, and are very time consuming. In wastewater treatment plants, measurement of TP is usually only made on a daily basis. Hence any aberrations from optimal conditions are not detected until well after the event, and remedial or preventative action is precluded.

The use of on-line monitoring systems is therefore highly desirable, and this paper described two flow injection digestion approaches which have been developed for determination of TP in wastewater treatment, effluents and fresh waters. The first, which involves an on-line microwave digestion procedure was developed specifically for wastewaters, while the second, which utilizes uv-photooxidation, was found to be more suitable for the analysis of fresh waters. Both methods compare favourably with TP results obtained by conventional batch methods.

On-line digestion and detection of TP is complicated by the anhomogeneous nature of the samples. Wastewaters, and even freshwaters, contain high concentrations of suspended particulate material and this must be removed prior to the detection stage. A miniaturized, self-priming tangential flow filtration (TFF) module has been developed for this purpose, and has been found to be suitable for extended use with the uv-photooxidation method. This TFF system uses a stream of air bubbles to generate turbulence, and minimize fouling of the membrane surface.

The advantages and limitations of the application of the TFF system to flow injection TP determination were discussed.

### Determination of cadmium in fertilizers and foodstuffs by flow-injection spectrophotometry

*José A. Gomes Neto, Instituto de Física e Química de S. Carlos, Universidade de São Paulo; H. Bergamin, F°, Elias A. G. Zagatto and Francisco J. Krug, Centro de Energia Nuclear na Agricultura, Universidade de São Paulo, 13416-000 Piracicaba SP (Brazil)*

Formation of the blue ternary complex between cadmium, iodide and Malachite Green was exploited for the spectrophotometric flow-injection determination of cadmium. Addition order and concentrations of reagents, interaction time between iodide and Malachite Green, flow rates, pH for ternary complex formation, temperature, surfactant addition, and presence of potential interferent ions were studied. Linearity of the calibration curve was improved with cadmium addition.

Masking agents, kinetic discrimination, and ion-exchange separation were investigated, and a Chelex-100 resin mini-column (50–100 mesh) was elected for in-line interferent removal in foodstuff analysis. Sampling rate and detection limit depends on the selected conditions for concentration and elution. Results are reproducible (r.s.d. about 0·02) and comparable with graphite furnace atomic absorption spectrometry.

The system for fertilizer analysis does not includes ion-exchange separation, handles about 90 samples per hour (10–100 µg 1-1), and is characterized by a detection limit of 2 µg Cd 1-1. Precise results (r.s.d. usually 0·005) are obtained. Accuracy was assessed against i.c.p.–a.e.s.

### Flow injection analysis: leaving its teen-years and maturing. A personal reminiscence of its conception and early development

*Elo Harald Hansen, Chemistry Department A, The Technical University of Denmark, Building 207, DK 2800, Lyngby, Denmark*

The occurrence of Flow Analysis VI in Toledo marks almost to date the very time when my good friend and former colleague Professor Jaromir (Jarda) Ruzicka and I, 20 years ago in Denmark, made the first exciting experiments of the methodology for which we coined the name 'Flow Injection Analysis' (FIA). As most of us probably have experienced when passing a round birthday, such an occasion usually gives rise to reflections over the time and events gone by. And since FIA has played such an important role in our professional lives, and proved to be a viable analytical tool for others as well, it came very naturally to me at the 20th birthday of FIA to dig into the past and make some recollections over the conception of FIA, and to make some speculations as to its rapid development as reflected in the number of FIA publications which have appeared in international periodicals over the past two decades.

The first FIA paper was published in *Analytica Chimica Acta* in 1975, and after an initial lag phase FIA in earnest gained momentum. Thus, by 1979, at the occasion of the first flow analysis meeting in Amsterdam, about 80 publications had appeared. When we wrote our first book on FIA in 1980, the number had just managed to pass the magic mark of 100. Undoubtedly, spurned by the early flow analysis conferences the growth continued to a rapid rate, and at the time of FA III in Birmingham in 1985, slightly more than 800 papers had been published—and notably languages other than English had become represented on the list. By Spring 1987, when we submitted the second edition of our book, the number had swelled to well over 1400. And this explosive growth has continued ever since, reaching by early 1994 approximately 5300 FIA publications. Besides, more than 15 monographs have appeared, which on one hand demonstrates the impact FIA has had on modern analytical chemistry, and on the other hand scaringly proved us almost clairvoyant when we wrote in the second edition of our book: 'It seems as if the spirits have released a tidal wave of FIA publications far beyond imagination, the flooding power of which is rapidly undermining our ability to write about it'.

This lecture described the birth and early development of FIA. Possible (and, indeed, impossible) explanations for the ready acceptance of FIA as an analytical chemical tool were discussed.

